# Environmental and occupational respiratory diseases – 1059. Lung exposure to TiO_2_ induces eosinophilic airway inflammation in the rabbits

**DOI:** 10.1186/1939-4551-6-S1-P57

**Published:** 2013-04-23

**Authors:** Gil-Soon Choi, Hee-Kyoo Kim, Chul-Ho Ok

**Affiliations:** 1 Department of Internal Medicine, College of Medicine, Kosin University, Busan, South Korea

## Background

Titanium dioxide (TiO2) nanoparticels (NPs), one of the most abundantly utilized nanomaterials, was widely used in industry, cosmetics, and biomedicine. There have been reported that TiO2 NPs aggravates respiratory symptoms by induce pulmonary inflammation. However, the mechanisms of these effects have not been extensively studied yet. We aimed to investigate inflammation in rabbit lung following an intratracheal instillation.

## Methods

To understand lung inflammation after TiO2 NPs exposure, TiO2 NPs were instilled into one lung of rabbits at fixed dose of 10, 50, 250ug, respectively. Bronchoalveolar lavage fluid (BALF) was collected before (baseline), at 1 and 24hr after TiO2 NPs intratracheal instillation. Changes of inflammatory cell in BALFs were measured. After BAL processing, lung histological assay were carried out at 24hr after TiO2 NPs instillation. For lung image analysis, chest computer-tomography scan was performed at 1, 24hr after instilled TiO2 NPs and normal saline into each lung.

## Results

The eosinophil count in BALF were significantly increased at 1 and 24hr after TiO2 NPs instillation. Furthermore, TiO2 NPs induced a dose dependent increase of eosinophils in BALF. No significant differences in any of the other inflammatory cell counts (e.g. neutrophils and lymphocytes) were detected. In the lung tissue, severe eosinphilic inflammation and macrophage apoptosis with hemorrhage was observed at 24hr TiO2 NPs postinstillation. Radiologic finding showed ground glass opacity in both lung at the 1hr after TiO2 NPs and normal saline (control) postinstillation. Although control lung showed complete resolution at 24hr, other lung exposed by TiO2 NPs was persistently showed ground glass opacity until 24hr.

## Conclusions

We confirmed that TiO2 NPs induced lung inflammation in rabbits, and this process may be associated with eosinophilic inflammation.

